# Whole-genome Analysis Reveals Contrasting Relationships Among Nuclear and Mitochondrial Genomes Between Three Sympatric Bat Species

**DOI:** 10.1093/gbe/evac175

**Published:** 2022-12-22

**Authors:** Veronika N Laine, Tiina Sävilammi, Niklas Wahlberg, Katarina Meramo, Gonzalo Ossa, Joseph S Johnson, Anna S Blomberg, Aidyn B Yeszhanov, Veronica Yung, Steve Paterson, Thomas M Lilley

**Affiliations:** BatLab Finland, Finnish Museum of Natural History, University of Helsinki, Helsinki, Finland; Department of Biology, University of Turku, Turku, Finland; Department of Biological and Environmental Science, University of Jyväskylä, Jyväskylä, Finland; Department of Biology, Lund University, Lund, Sweden; BatLab Finland, Finnish Museum of Natural History, University of Helsinki, Helsinki, Finland; ConserBat EIRL, San Fabian, Chile; Asociación Murciélagos de Chile Pinüike, Santiago, Chile; Department of Biological Sciences, University of Cincinnati, Cincinnati, Ohio, USA; Department of Biology, University of Turku, Turku, Finland; Institute of Zoology of the Ministry of Science and Education of the Republic of Kazakhstan, Almaty, Kazakhstan; Departamento Laboratorio Biomédico, Instituto de Salud Pública de Chile, Santiago, Chile; Evolution, Ecology and Behaviour, Institute of Infection, Veterinary and Ecological Sciences, University of Liverpool, Liverpool, UK; BatLab Finland, Finnish Museum of Natural History, University of Helsinki, Helsinki, Finland

**Keywords:** bats, phylogeny, nuclear DNA, mitochondrial DNA, gene flow, speciation

## Abstract

Understanding mechanisms involved in speciation can be challenging, especially when hybridization or introgression blurs species boundaries. In bats, resolving relationships of some closely related groups has proved difficult due subtle interspecific variation both in morphometrics and molecular data sets. The endemic South American *Histiotus* bats, currently considered a subgenus of *Eptesicus*, harbor unresolved phylogenetic relationships and of those is a trio consisting of two closely related species: *Eptesicus (Histiotus) macrotus* and *Eptesicus (Histiotus) montanus*, and their relationship with a third, *Eptesicus (Histiotus) magellanicus*. The three sympatric species bear marked resemblance to each other, but can be differentiated morphologically. Furthermore, previous studies have been unable to differentiate the species from each other at a molecular level. In order to disentangle the phylogenetic relationships of these species, we examined the differentiation patterns and evolutionary history of the three *Eptesicus* (*H.*) species at the whole-genome level. The nuclear DNA statistics between the species suggest strong gene flow and recent hybridization between *E. (H.) montanus* and *E. (H.) macrotus*, whereas *E. (H.) magellanicus* shows a higher degree of isolation. In contrast, mitochondrial DNA shows a closer relationship between *E. (H.) magellanicus* and *E. (H.) montanus*. Opposing patterns in mtDNA and nuclear markers are often due to differences in dispersal, and here it could be both as a result of isolation in refugia during the last glacial maximum and female philopatry and male-biased dispersal. In conclusion, this study shows the importance of both the nuclear and mitochondrial DNA in resolving phylogenetic relationships and species histories.

**Significance**

The status of the South American *Histiotus* bat species is controversial and their phylogenetic relationships have been so far unresolved. Our study shows that *Histiotus* have experienced a radiation after the relatively recent colonization to the Neotropics. This is the first study to investigate the systematic relationships between *Histiotus* bats at a whole-genome level and more importantly, highlights importance of inspecting both mitochondrial and nuclear DNA evidence in understanding the evolutionary history of species.

## Introduction

The degree of taxonomic resolution available to uncover global biodiversity patterns has increased by orders of magnitude with the introduction of more advanced molecular methods. These allow for more precise documentation of life on Earth at a time of rapid loss of biodiversity (IPBES 2022). However, advanced approaches also add to the complexity of interpreting the results and not allowing for standard species delimitation processes. For instance, a volume of studies over the last decades have heavily relied on mitochondrial sequences to infer phylogenetic relationships between species (Bargelloni et al. 2000; Bibi 2013; Cameron 2014). With the advent of whole-genome sequencing, it has become evident that phylogenies constructed using mitochondrial data may tell a different story to those using nuclear data (Platt et al. 2018). Therefore, disentangling current relationships between related species requires an understanding of processes that have affected, and continue to affect, divergence. As an example, the quaternary glaciations have had a profound effect on historical distribution and gene flow promoting genetic divergence among species and populations (Hewitt 2004; Weir and Schluter 2004).

With c. 1,400 species described so far, bats (Chiroptera) form a group containing broad ecological and morphological diversity (Simmons and Cirranello 2019). However, resolving the systematic relationships of some groups using morphometrics or molecular methods has proved to be challenging (Jones et al. 2002; Van Den Bussche and Lack 2013). One example is represented by the complex vespertilionid genus *Eptesicus* (Rafinesque, 1820), which exhibits only subtle interspecific variation. *Eptesicus* is one of the larger genera of Vespertilionidae, with over 30 species described to date (Wilson and Mittermeier 2019). The genus is found on all continents except Antarctica, with the greatest diversity found in South America (Wilson and Reeder 2005). In South America, *Eptesicus* comprises 16 species so far (Díaz et al. 2019). *Eptesicus* inhabit almost every ecosystem on the continent, from the high altitudes of the Andes (Baker 1974), to the coastal Atlantic Forest of Brazil (Miranda et al. 2007), the semiarid pampas of Argentina (Barquez et al. 2012), the Atacama desert (Ossa et al. 2014), and the temperate forest of southern Chile (Altamirano et al. 2017).

*Histiotus* is considered a subgenus of *Eptesicus* (Hoofer and Van den Bussche 2003; Roehrs et al. 2010; Amador et al. 2018; Simmons and Cirranello 2019). Divergence from genus *Eptesicus* sensu stricto has taken place rather recently, with a Cytochrome b (*CYTB*)-dated tree by Giménez et al. (2019) suggesting a split with *Eptesicus* occurring roughly 5 Ma. The development of the Andean region, glacial cycles, and associated glacial refugia most likely contributed to early diversification of *Histiotus* (Díaz et al. 2019; Giménez et al. 2019). The subgenus, with suggested taxonomic status of *Eptesicus* (*H*.), is endemic to South America and includes eight currently recognized species. The status of some of these species is controversial and their phylogenetic relationships remain unresolved (Giménez et al. 2019).

**Fig. 1.**
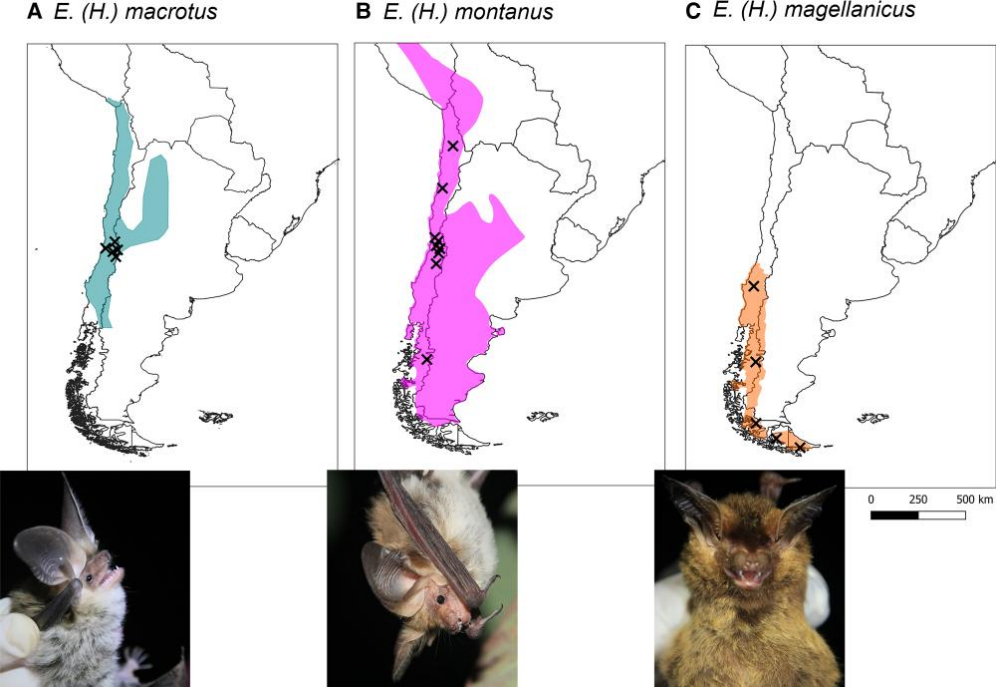
The map of South America with *Eptesicus* (*H.*) species distribution ranges and sampling locations. (*A*) *E. (H.) macrotus* (type locality Antuco, Chile, Poeppig 1835), (*B*) *E. (H.) montanus* (type locality Cordillere von Santiago, Chile, Philippi and Landbeck 1861, most northern sample Hmon_436), and (*C*) *E. (H.) magellanicus* type locality Agellan Strait, Chile, Philippi 1866, most northern sample Hmag_1501). Distributions according to Marsh et al. (2022) with modifications by authors based on own records.

One unresolved species trio consists of two closely related species: the big-eared brown bat *Eptesicus (Histiotus) macrotus* and the small big-eared brown bat *Eptesicus (Histiotus) montanus*, and their relationship with a third, the southern big-eared brown bat *Eptesicus (Histiotus) magellanicus*. The species resemble each other, but can be differentiated in morphospace, with *E. (H.) magellanicus* segregating from other *Eptesicus (H.)* species by its darker pelage, wing membranes, and pinnae, and smaller ears on average (Barquez et al. 1993, 1999; Giménez et al. 2012; Giménez and Giannini 2017). As for *E.* (*H.) montanus* and *E. (H.) macrotus*, the species can be distinguished from each other by the larger ears of the latter (males 29.2 ± 1.8 [*n* = 9], females 31.1 ± 2.4 [*n* = 31]), compared with the former (males 25.9 ± 3.1 [*n* = 28], females 27.3 ± 2.9 [*n* = 80]). Furthermore, *E. (H.) macrotus* is generally slightly larger with a forearm length of males at 48.8 ± 1.0 mm (*n* = 9), and females at 49.6 ± 1.3 (*n* = 31) compared with *E.* (*H.) montanus* with males at 46.6 ± 2.3 mm (*n* = 28) and females at 48.5 ± 2.0 mm (*n* = 80; Ossa G, personal data). All three species coexist in sympatry with overlaps in distribution range. However, the overlap between *E. (H.) magellanicus* and *E. (H.) macrotus* occurs only in the very northern part of the range of the former (see fig. 1, Koopman 1967; Giménez et al. 2012; Rodriguez-San Pedro et al. 2016). In the past, the taxonomic resolution between these three species has been coarse (Feijó et al. 2015). Until 1999, *E. (H.) magellanicus* was classified as a subspecies of *E. (H.) montanus* (Barquez et al. 1999), and another member of the subgenus, *E.(H.) laephotis*, was classified as a subspecies of *E. (H.) macrotus* until 2005 (Simmons 2005). However, their mutual identity as distinct species has been questioned in a recent study based on molecular methods (Giménez et al. 2019).

Here, to our knowledge for the first time, we attempt to disentangle the phylogenetic relationship within three species of austral *Eptesicus* (*H.*) using a whole-genome approach. Previous work on their systematic status at the mitochondrial level suggests distinct species status of *E. (H.) magellanicus* despite of its sympatric coexistence with *E. (H.) macrotus* and *E. (H.) montanus* (Giménez et al. 2019). However, no internal resolution could be achieved between *E. (H.) macrotus* and *E. (H.) montanus*. More specifically, here we (1) explore the genetic relationships of the *Eptesicus* (*H.*) bat assemblage in Patagonia using whole-genome data and (2) examine how patterns of segregation between species are manifested in nuclear versus mitochondrial DNA (mtDNA), and finally, (3) improve the taxonomic resolution of the species complex.

## Results

### Mapping and Genotype Likelihood Calling

Our samples mapped back to the reference genome with an average rate of 98.0% (supplementary table S1, Supplementary Material online). Altogether 14,654,572 genotype likelihood sites were called from the *Eptesicus* (*H.*) species and were left with 5,900,898 sites after LD pruning. With the inclusion of *Eptesicus bottae* and *Myotis* species, a total of 9,943,522 sites were called.

### Population Structure and Demography in *Eptesicus* (H.)

**Fig. 2.**
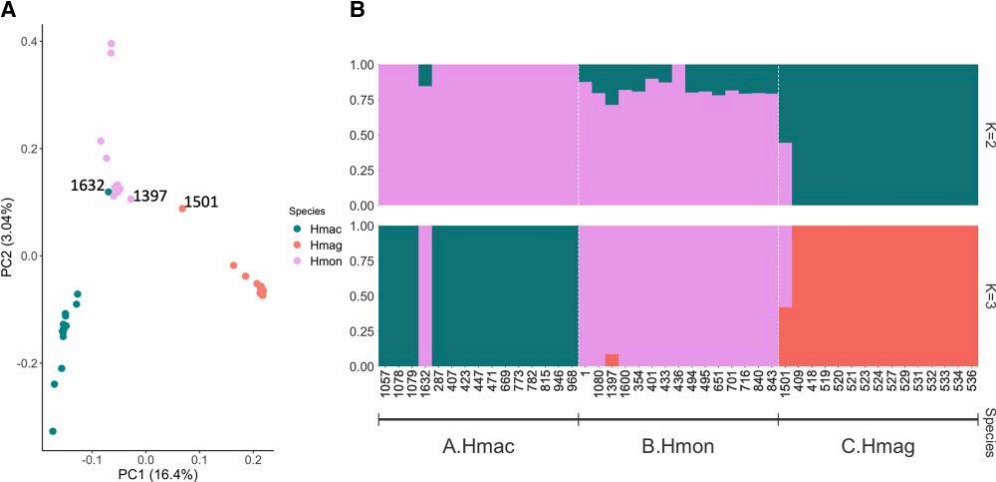
(*A*) PCA showing the first and second PCs. The proportion of genetic variance captured by each component is indicated between parentheses. (*B*) Ancestry proportions for the *Eptesicus* (*H.*) individuals inferred in NGSAdmix with *K* = 2 and 3.

The results from the principal component analyses are illustrated in figure 2*A*. The first principal component explained 16.4% of the genetic variation, separating Hmag from Hmac and Hmon. The second principal component explained 3.04% of the variation and separated Hmac and Hmon. A strong outlier, Hmac_1632, was detected. This sample, morphologically identified as Hmac, clustered with Hmon in the PCA.

Admixture was tested using two approaches. *K* = 2 was identified as the highest level of structure using the Evanno method, whereas *K* = 3 had the highest Pr(*K* = *k*) value using the STRUCTURE method. Using *K* = 2, Hmac and Hmag formed distinct groups, whereas Hmon was a mixture of the two (fig. 2*B*). With *K* = 3, each *Eptesicus* (*H.*) species represented a distinct group, but three individuals (Hmac_1632, Hmon_1397, and Hmag_1501) from each species exhibited some degree of admixture of which Hmac_1632, already mentioned in previous paragraph, was a clear outlier and showed distinctive Hmon pattern (fig. 2*B*).

### Diversity and Evolutionary History

The pairwise Fst-values between Hmac and Hmon were 0.0717, 0.1434 between Hmag and Hmon, and 0.2168 between Hmac and Hmag. Nucleotide diversity, Theta Watterson, and Tajima's *D* were similar between *Eptesicus* (*H.*) species (supplementary fig. S1, Supplementary Material online).

### Genetic Introgression and Nuclear DNA Phylogeny

**Fig. 3.**
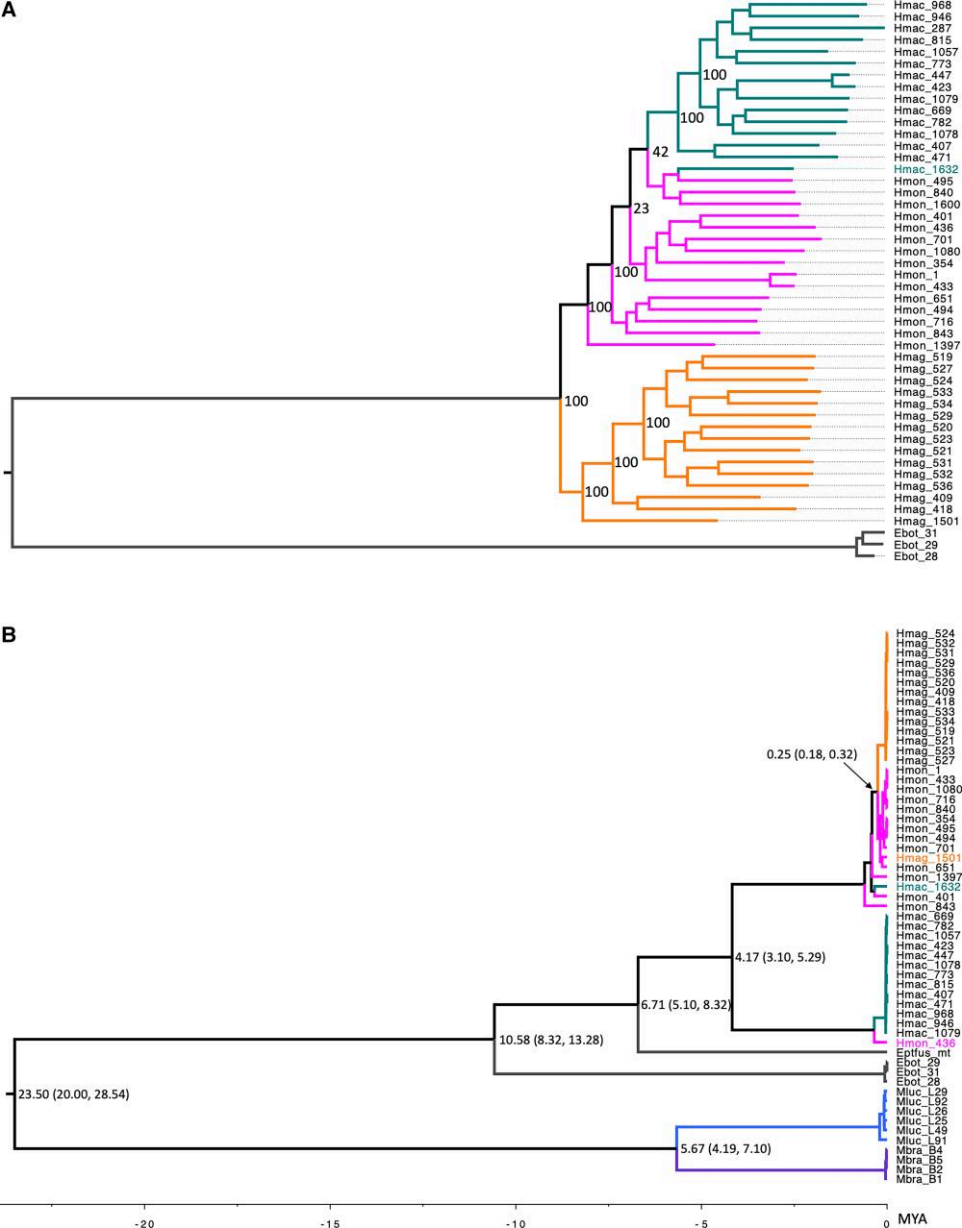
(*A*) Nuclear species tree derived from 8,511,209 SNPs by using maximum likelihood method with bootstrap values indicated at the nodes and (*B*) mitochondrial species tree using 13 protein coding genes built with BEAST2 with the estimated divergence times (Ma) given at the nodes, and the 95% confidence intervals in parentheses.

D-statistics showed significant gene flow between Hmac and Hmon (supplementary Table S2, Supplementary Material online) by having significant positive *D*-value (*Z* > 3, and *P*-value = 0). These two also formed a monophyletic group together both in the neighbor joining and maximum likelihood trees separating them from Hmag (fig. 3*A*, supplementary fig. S2, Supplementary Material online). However, with most of the variation occurring at the individual level, clear branching is not evident in the neighbor joining tree, but clear separation with high bootstrap levels are observed in the maximum likelihood tree. Once more, Hmac_1632 clustered with Hmon in both trees.

### Mitochondrial DNA Assembly and Phylogeny

We assembled 57 individual mitochondria from seven different species. Our alignment consisted of 18,049 nucleotide sites of which 12,126 (= 67.18% of all sites) were invariable. After extracting the protein coding and rRNA genes, the number of parsimony informative sites was 4,428 and the number of distinct site patterns was 1,610. In IQ-TREE, the best-fit partitioning outcomes were group 1: *ND1*, *ND2*, *ND3*, *ND4L*, *ND4*, *ND5*, *ND6*, *ATP6*, *ATP8,* group 2: *COX1*, *COX2*, *COX3*, *CYTB*, and group 3: 12S, 16S. The best-fit models according to BIC were for group 1 TIM + F + I, for group 2 TPM2 + F + G4, and for group 3 TIM2 + F + G4. The derived maximum likelihood tree is presented in supplementary figure S3, Supplementary Material online with bootstrap values. Molecular divergence times obtained from BEAST2 were similar to the set priors with the exception of the Eptfus and *Eptesicus* (*H.*) branch being estimated at 6.71 Ma (calibration 3.87 Ma). The estimated divergence time for Hmac and Hmon/mag, which has not been presented in previous studies, was 4.17 Ma and the divergence time between Hmag and Hmon was estimated at 0.25 Ma (as opposed to 0.74 Ma in Upham et al. 2019). The BEAST2 tree with divergence times are presented in figure 3*B*.

In general, none of the three species were found to be monophyletic using mitochondrial data. Once more, Hmac_1632 groups with Hmon. We also observed two other individuals (Hmag_1501 with Hmon and Hmon_436 with Hmac), which were not within their corresponding species branch. Hmag_1501 was also an outlier in the admixture analyses (*K* = 3). However, Hmon_436 was not an outlier in any of the nuclear analyses. The mitochondrial trees also differ from the nuclear trees with regards to the relationships between the *Eptesicus* (*H.*) species. Based on the mitochondrial genes Hmon and Hmag appear to be very closely related to each other and quite distant to Hmac (divergence time 4.40 Ma). In contrast, the nuclear DNA admixture and evolutionary analyses suggest that Hmon and Hmac are more closely related to each other than either of these are to Hmag (figs. 2 and 3*A*).

### *CYTB* Gene Tree Comparison

The *CYTB* tree showed the same structure as when looking at all the 13 mitochondrial protein coding genes in the previous section, Hmag and Hmon are more closely related and Hmac more distant from the previous two (supplementary fig. S4, Supplementary Material online). However, the Hmag individuals from Giménez et al. (2019) study cluster well with our Hmag individuals whereas all their Hmac and Hmon samples group with our Hmac individuals.

## Discussion

The results of the study clearly present discordance between nuclear and mtDNA patterns in the three species studied. All the nuclear DNA statistics between the species suggest strong gene flow and some degree of hybridization between *E. (H.) montanus* and *E. (H.) macrotus*, whereas *E. (H.) magellanicus* shows a higher degree of isolation. The test for ancient admixture, calculated with the *D*-statistics, suggests *E. (H.) montanus* and *E. (H.*) *macrotus* experience contemporary gene flow between the populations. In contrast, mtDNA shows *E. (H.) magellanicus* grouping with *E. (H.) montanus* in a paraphyletic assemblage, with high bootstrap values indicative of high confidence for each of the clades, which suggests past introgression amongst the species.

### Nuclear Evidence

Our whole-genome approach surprisingly revealed that *E. (H.) montanus* and *E. (H.) macrotus* exhibit a high degree of gene flow between our sampled populations, with *E. (H.) magellanicus* being significantly genetically isolated. This result was supported by not only the population structure analysis, but also the Fst and *D*-statistics tests, adding weight and certainty to the findings. These findings are in sharp contrast to those presented by Giménez et al (2019) who suggested no resolution of phylogenetic relationships within *Eptesicus* (*H.*) based on nuclear DNA. However, here, a single nuclear intron (thyrotropin, *THY*) was used, which appears to have very little variation across the entire genus *Eptesicus*. In our study, the nuclear species tree based on SNPs suggests that *E. (H.) magellanicus* is monophyletic (including Hmag_1501), whereas *E. (H.) montanus* is paraphyletic with regards to *E. (H.) macrotus*, and Hmac_1632 renders *E. (H.) macrotus* paraphyletic with regards to *E. (H.) montanus*. The test for ancient admixture suggests hybridization between *E. (H.) montanus* and *E. (H.) macrotus* is contemporary and may present an example of speciation with gene flow. This is certainly feasible as the karyotypes of all *Eptesicus* (*H.*) are identical [2*n*] = 50; fundamental number = 48, with acrocentric autosomes only (Williams and Mares 1978). Furthermore, the species distribution of *E. (H.) montanus* and *E. (H.) macrotus* show a more significant overlap with each other than with *E. (H.) magellanicus*.

However, the discrepancy at the admixture analysis between the best K methods and low genetic variation explained in the PCA revealed that the analyses have difficulty in separating the species. This considered, future research could benefit from a wider geographic sampling of the three species using deeper coverage sequencing to better understand how both introgression and hybridization maybe affecting these taxa.

### Mitochondrial Evidence

The mitochondrial alignments for the three species present a differing narrative to the nuclear data, with high bootstrap values indicating clear definition between the mitochondrial lineages. However, whereas *E. (H.) montanus* and *E. (H.) macrotus* were nested next to each other in the nuclear data, it is *E. (H.) magellanicus* and *E. (H.) montanus* that appear more closely related to each other using mitochondrial data. The results also differ from those presented by Giménez et al. (2019), in which *E. (H.) magellanicus* was the first to diverge followed by no internal resolution in a clade containing *E. (H.) montanus* and E*. (H.) macrotus*. This led the authors to believe local hybridization or introgression was the causative agent. The study utilized a single mitochondrial gene (*CYTB*), whereas the present study included all the protein coding mitochondrial gene alignments, increasing the amount of data used to construct the phylogenies by orders of magnitude. Furthermore, when comparing our *CYTB* sequences to Giménez et al. (2019), the *E. (H.) magellanicus* from both data sets clustered together. However, both *E. (H.) montanus* and *E. (H.) macrotus* from Giménez et al. are clearly clustering with our E*. (H.) macrotus*, suggesting misidentification may have occurred in the Giménez et al. study. However, we can also not disclose the possibility that wider geographical sampling may reveal a more complex sorting pattern of mitochondrial haplotypes, which would also explain conflict between the existing and previous results. Furthermore, phylogenic discordances due to incomplete lineage sorting may become more evident when using single genes in phylogenetic analyses (Wang et al. 2018; Lopes et al. 2021). Thus, future studies should also concentrate on whole-genome sequences to reveal true relationship between these species.

We found evidence of hybridization and introgression operating at different time scales by studying both the nuclear admixture and mitochondrial haplotypes. The mitochondrial haplotype of Hmac_1632 is most closely related to *E. (H.) montanus*, as is the haplotype of Hmag_1501. Hmac_1632, which is morphologically identified as *E. (H.) macrotus*, showed some admixture at *K* = 2, but appeared to be purely *E. (H.) montanus* genetically at *K* = 3, suggesting that the interbreeding took place some generations ago or the individual has been misidentified. Hmag_1501 also showed admixture when analyzed with *K* = 2 and *K* = 3, suggesting very recent hybridization, possibly even F1. Although the mitochondrial haplotype is not identical to any of the sampled *E. (H.) montanus* haplotypes, it might be possible to find this very haplotype in the extant populations of *E. (H.) montanus* with further sampling.

Introgression dating further back may have been observed in Hmon_436 which has a mitochondrial haplotype close to the *E. (H.) macrotus* haplotypes but does not show any evidence of admixture at nuclear level. Thus, this individual may be the remnant of a more ancient interbreeding event, where a female *E. (H.) macrotus* would have hybridized with a male *E. (H.) montanus*. The subsequent offspring would have then back-crossed with the *E. (H.) montanus* population for several generations, leaving only the mitochondrial haplotype of *E. (H.) macrotus* as evidence of this event. The mitochondrial haplotype of Hmon_436 is also somewhat diverged from the rest of the *E. (H.) macrotus* haplotypes, which is suggestive of sometime (c. 0.8 Ma according to divergence times) passing as the transfer of the *E. (H.) macrotus* haplotype to the *E. (H.) montanus* population. Based on the times of divergence calculated from the mitochondrial genomes, the main mitochondrial haplotypes of *E. (H.) montanus* and *E. (H.) macrotus* appear to have diverged from each other over 4 Ma. Even though the interbreeding event appears to have taken place long enough ago that the nuclear genome does not hold any remnants of this, the branch lengths suggest that the events occurred less than 1 Ma (fig. 3*B*).

Differences between nuclear and mtDNA have been documented in several species (Toews and Brelsford 2012; Platt et al. 2018) and observational and simulation studies have shown that the opposite patterns of introgression at mtDNA and nuclear markers is often due to differences in dispersal behavior (Petit and Excoffier 2009). The rate of introgression is often negatively correlated with the rate of intraspecific gene flow and in cases where dispersal is male biased, the lower gene flow associated with the maternally inherited mtDNA could account for a higher rate of introgression (Petit and Excoffier 2009).

Our observations of the relatively distant relationships between the majority of *E. (H.) macrotus* and *E. (H.) montanus* haplotypes in the mitochondrial tree (fig. 3*B*) and the presence of admixture between these two species in the nuclear genome (figs. 2 and 3*A*) provide molecular evidence likely reflecting female philopatry and male-biased dispersal. For many temperate, non-migratory bat species, dispersal is primarily male driven (Burland and Wilmer 2001; Moussy et al. 2013). In some cases, the bias toward male dispersal may be extreme (Kerth et al. 2002). Although there are some exceptions in the dispersal strategies of temperate bats (Entwistle et al. 2000), the strategies are much less variable than among the tropical species where dispersal of both sexes as well as sex-specific dispersal of either males or females has also been reported (McCracken and Bradbury 1981; Wilkinson 1985; Storz et al. 2001; Ortega et al. 2003; Dechmann et al. 2007; Nagy et al. 2007). So far, no reports exist on the dispersal behavior of the focal species in our study (Díaz et al. 2019). However, they bear rather close affinity to *E. fuscus*, in which even female dispersal and gene flow have been observed (Vonhof et al. 2008), a behavior however lacking in the Palearctic counterpart, *Eptesicus nilssonii* (Suominen et al. 2022). Therefore, we cannot simply assume one or the other for our focal species. This highlights the importance of rigorous genomic sampling at a greater geographic scale and a more complete understanding of the natural history to disentangle the processes responsible for the discordant patterns of genome evolution found in our data. Bridging the gap between genetic information, ecology, natural history, and theory is of tremendous importance in understanding the effects of a variety of evolutionary processes that may be at play here (Lawson Handley and Perrin 2007; Toews and Brelsford 2012).

In *Myotis* species, differences between mtDNA and nuclear DNA variation are common. This suggests that lineage sorting, reticulation, and introgression have likely influenced the genomes of *Myotis* (Platt et al. 2018). For example, the mitochondrial genome of the *Myotis blythii* has been replaced by that of *Myotis myotis*. In contrast, both species are differentiated at nuclear markers. Both species have also a male-biased gene flow thus this agrees with the expectation that mtDNA should introgress more readily than biparentally inherited nuclear DNA (Berthier et al. 2006). Discrepancy between mtDNA and nuclear DNA has also been shown in other bat species such as Asian *Rhinolophus* (Mao et al. 2010), the African *Scotophilus* (Vallo et al. 2011) and in the Old World leaf-nosed bats (Hipposideridae) (Patterson et al. 2020). Similarly, mtDNA introgression has also reported in other *Eptesicus* (Artyushin et al. 2009; Juste et al. 2013). In *Eptesicus serotinus*, two different mtDNA lineages have been observed, one similar to *E. nilssonii* and the other distinct. Following the theory of Currat et al. (2008) where the direction of introgression is preferentially from local species toward invading, interbreeding between these two species could have occurred asymmetrically during last glacial maximum (LGM). With respect to the biogeography of our focal species, glaciers covered much of Tierra del Fuego and Patagonia during the LGM (Rabassa et al. 2011). Refugia for this period have been placed to the north of latitude 40 to the west of the Andes, whereas on the eastern side, refugia were located on the present submarine shelf perhaps all the way down to the latitude of Isla de los Estados at 54°S (Fraser et al. 2012). One plausible explanation for the pattern seen here is that *E. (H.) magellanicus* resided through the LGM at refugia situated to the east of the Andes, whereas *E. (H.) montanus* and *E. (H.) macrotus* shared refugia to the north where speciation with gene flow could have occurred. Secondary contact between *E. (H.) montanus* and *E. (H.) magellanicus* after the LGM would explain introgression of mtDNA in these species. However, to fully understand the effect of these processes on contemporary populations, more genetic sampling at a broader geographical scale is needed.

## Conclusion

To our knowledge, this is the first study to investigate the systematic relationships between *Eptesicus* (*H.*) bats at a whole-genome level. This approach allows us a unique insight into the processes that have shaped the speciation evolution of this austral bat subgenus. Despite its evident roots deep in the *Eptesicus* -clade, the *Eptesicus* (*H.*) bats of South America have experienced a radiation after the relatively recent vespertilionid colonization of the Neotropics, with evidence of their speciation being still incomplete. Similar processes have been recorded in other vespertilionids, such as the *Myotis* (Morales and Carstens 2018). Moreover, our study highlights importance of inspecting both mitochondrial and nuclear DNA evidence to better understand the evolutionary history of species, as well as the applicability of genome likelihood.

## Materials and Methods

### Sample Collection, DNA Extraction and Sequencing

We obtained 45 skin samples, 15 of each, of three species, *E. (H.) montanus* (from here on Hmon), *E. (H.) macrotus* (from here on Hmac), and *E. (H.) magellanicus* (from here on Hmag) from two sources. Fourteen samples were obtained by mist netting during November and December 2017 at two localities: Chicauma (33°S 70°W) and Karukinka Reserve (54°S 68°W), respectively. Samples were obtained with disposable biopsy punches (5 mm) from captured individuals, which were released at the capture site. Furthermore, 31 samples were obtained from the Public Health Institute of Chile, from deceased individuals that had been sent by the public to the rabies laboratory for monitoring. Samples were collected from the plagiopatagium using sterile scalpel and stored in 1.5 ml tubes with 95% EtOH at −20 °C until further analysis. Because some samples were collected from live individuals and others from carcasses, we have not included data on morphology, as these are not comparable.

We also used three species (*Eptesicus bottae* [Ebot], *Myotis brandtii* [Mbra], and *Myotis lucifugus* [Mluc]) as outgroups for our phylogenetic analyses. The three individuals of *E. bottae* were sampled at Birlik village, Kazakhstan as a part of field work associated with the project "BR10965224-OT-22 Development of a cadastre of the fauna of the Northern Tien-Shan to preserve its genetic diversity". The four individuals of *M. brandtii* were collected from Russia, Finland, Germany, and Latvia. The six *M. lucifugus* individuals were collected from the United States. The outgroup individuals were caught with mistnets, sampled and released in various third party projects. Please see supplementary table S1, Supplementary Material online for complete list of samples used.

The DNA for each species was extracted using QIAmp DNA Mini Kits (Qiagen, Hilden, Germany) and stored the DNA at −80 °C. The sequencing and read trimming was conducted at the University of Liverpool Centre for Genomic Research. TruSeq Nano libraries with a 350 bp insert size were prepared from all samples and run on a HiSeq4000 platform to generate 2× 150 bp reads. Adapter sequences were removed from all sequenced reads with Cutadapt v1.2.1 (Martin 2011) and trimmed with Sickle 1.200 (Joshi and Fass 2011) with a minimum quality score of 20 and then used as an input for the analysis. The low-coverage whole-genome sequencing of *Eptesicus* (*H*). samples provided ∼183 Gbp of sequence data (∼1.9× coverage) for each individual, whereas the whole-genome sequencing of *M. brandtii* and *M. lucifugus* resulted in the average of 14.1× and 7.0× coverage per sample, respectively (supplementary table S1, Supplementary Material online).

### Read Mapping and Genotype Likelihood Calling

All species were mapped against *Eptesicus fuscus* genome (GCA_000308155.1 EptFus1.0) using bwa v. 0.7.17 (Li and Durbin 2009) mem command with slightly relaxed read mapping parameters -B 3 (mismatch penalty), -O 5 (gap open penalty), and -k 15 (minimum seed length) to allow mapping the reads of a closely related species. Due to low-coverage sequencing, genotype likelihoods were called instead of single nucleotide polymorphisms (SNPs) with ANGSD 0.935 (Li 2011; Korneliussen et al. 2014) from the bam files with the following specifications and filters: -GL 1 -ref GCA_000308155.1_EptFus1.0_genomic.fna -doGlf 2 -doMajorMinor 1 -doMaf 1 -uniqueOnly 1 -remove_bads 1 -only_proper_pairs 1 -trim 0 -C 50 -baq 1 -setMaxDepth 100 -minMapQ 20 -minQ 20 -minMaf 0.05 -minInd 30 -doCounts. This was done for only *Eptesicus* (*H.*) and all the species separately.

To minimize nonindependence due to linkage, genotype likelihood sites were pruned with ngsLD 1.1.1 (Fox et al. 2019) with maximum distance of 100 kb. LD decay was inspected and showed LD was negligible after a distance of 5 kb. LD pruning was run with default settings (−max_kb_dist 5 –min_weight 0.5) to obtain unlinked sites. Population structure analyses (PCA and admixture) were run on the set of unlinked sites and pruning was conducted only for *Eptesicus* (*H.*) species. All the other analyses (demographic history or selection) were run on the full sets of sites.

### *Eptesicus* (H.) Population Structure and Demography

Population structure was first assessed by running a principal component analysis using PCAngsd (Meisner and Albrechtsen 2018) on the pruned genotype likelihood data. The proportion of variance explained by each component was calculated with R function eigen.

An admixture analysis was run with NgsAdmix v.32 (Skotte et al. 2013), using the pruned genotype likelihoods estimated with ANGSD. NgsAdmix was run 10 times for each *K*-value between 1 and 3, using default values. The highest level of structure, that is the best *K* was identified using the Evanno method (Evanno et al. 2005) with CLUMPAK (Kopelman et al. 2015) using the likelihood values from each run. CLUMPAK also provides the best *K* calculated using the STRUCTURE method (Pritchard et al. 2000).

### *Eptesicus* (H.) Genetic Diversity and Evolutionary History

Nucleotide diversity, Theta Watterson, and Tajima's *D* were estimated for each *Eptesicus* (*H.*) species using ANGSD. First, the dosaf 1 function was used to calculate the site allele frequency spectrum likelihood (saf) for each species based on individual genotype likelihoods using the same specification as before but without maf -filter and lowering the -minInd to 10. Then, the realSFS function was used to optimize the saf and estimate the unfolded site frequency spectrum (SFS; Nielsen et al. 2012) adding -nSites 500,000,000 due to high memory consumption. Nucleotide diversity, Theta Watterson, and Tajima's *D* were calculated for each site with the commands saf2theta and thetaStat in ANGSD. To compute the 2D-SFS, the realSFS function was run on the saf files from each pair of species with the same -nSite value for the Fst estimation which was also done with ANGSD (realSFS fst stats -command). The outlier individual, Hmac_1632, was removed for both Fst estimation and *D*-statistics (see below).

### Genetic Introgression

Gene flow between species were estimated using Patterson's *D*-statistics calculated in ANGSD using the ABBABABA2 method (Soraggi et al. 2018). This test calculates the proportion of ABBA and BABA site patterns, and excess of either indicates admixture rather than incomplete lineage sorting (Durand et al. 2011). The three *E. bottae* individuals were used as an outgroup and all the possible combinations of *Eptesicus* (*H.*) species as H1, H2, and H3. We used the same quality specifications as in genotype likelihood calling but also restricting to sites with an SNP *P*-value <1.0 × 10^−6^ and taking only the first 1,000 largest scaffolds which covered 99% of the genome size and using the command -doAbbababa2 1 for the ABBABABA statistics. The *D*-values were called with the R-script estAvgError provided by ANGSD.

### Nuclear Phylogenetic Tree

A neighbor joining tree was constructed with the BioNJ tree building algorithm of FastME v.2.1.5 (Lefort et al. 2015), based on individual pairwise genetic distances estimated with ngsDist v.1.0.9 (Vieira et al. 2016) with bootstrapping (−n_boot_rep 100) using the ANGSD genotype likelihoods of all species. RAxML-NG v. 1.0.2 (Kozlov et al. 2019) was used to place the support values (command raxml-ng –support). FigTree v. 1.4.4 (http://tree.bio.ed.ac.uk/software/figtree/) was used to visualize the tree.

A maximum likelihood tree was constructed with an SNP panel. SNPs were called from the genotype likelihood file created above (all species) with BEAGLE Utilities program gprobs2beagle using minimum posterior probability of 0.8 (Browning 2013). Then beagle2vcf from BEAGLE Utilities was used to transform the file to a vcf-format, which was further transformed to PHYLIP-format with vcf2phylip v. 2.0 (Ortiz 2019) with minimum sample locus of 20. This provided 8,511,209 SNPs. The tree was constructed with IQ-TREE v. 2.1.4_beta (Minh et al. 2020) with model finder (Kalyaanamoorthy et al. 2017) and bootstrapping (1000) (-bb 1000 -m TEST). FigTree v. 1.4.4 was used to visualize the tree using Ebot as an outgroup.

### Mitochondrial DNA Assembly, Phylogeny and Molecular Timing

Whole mitochondria were assembled with GetOrganelle v. 1.7.5 (Jin et al. 2020) for all the samples using the Illumina reads. We used default animal mitochondria specifications (-k 21,45,65,85,105 and -F animal_mt) for most of the individuals. For some individuals, the final *k* was increased to 127 due to high coverage. Two individuals could not be assembled (Hmon_1600 and Hmac_287). The remaining 56 assembled individuals and an *E. fuscus* mitochondria from NCBI (MF143474.1) were aligned with Clustal Omega v. 1.2.4 (Sievers et al. 2011).

The Clustal alignment file was separated into 13 protein coding and two ribosomal RNA (rRNA) mitochondrial gene alignments based on the *E. fuscus* mitochondrial annotation and combined in one Nexus file. A consensus tree was built with IQ-TREE v. 2.1.4_beta with partitioning (Chernomor et al. 2016) and model finder with bootstrapping (1000) (-bb 1000 -m MFP + MERGE). FigTree v. 1.4.4 was used to visualize the tree.

We used BEAST2 v. 2.6.7 (Bouckaert et al. 2019) for molecular dating and BEAUti 2 to produce the run file for BEAST2. For this, we linked sites and clock models based on the IQ-TREE best partitioning outcome (group 1: *ND1*, *ND2*, *ND3*, *ND4L*, *ND4*, *ND5*, *ND6*, *ATP6*, *ATP8*; group2: *COX1*, *COX2*, *COX3*, *CYTB*; group 3: 12S, 16S). The trees were then linked as one. For both groups, the site model used was Gamma Site Model/GTR and clock model was Relaxed Clock Log Normal (Drummond et al. 2006). The priors used were Birth Death Model the calibration times from were obtained from Upham et al. (2019). The *Eptesicus*–*Myotis* split was given a uniform prior with a maximum 31 Ma and a minimum of 20 Ma, Myobra-Myoluc was assigned a normal prior of 7.67 Ma (±1.2 Ma), Eptfus-*Eptesicus* (*H.*) with a normal prior of 3.87 Ma (±1.7 Ma). We ran BEAST2 three times with chain length 50,000,000 and combined the trees with LogCombiner with 10% burnins and used TreeAnnotator for consensus tree. FigTree v. 1.4.4 was used to visualize the tree.

### CYTB Gene Tree Comparison

To compare our results with the previous *Eptesicus* (*H.*) phylogenetic study by Giménez et al. (2019), we obtained the mitochondrial *CYTB* gene sequences of the study from NCBI (NCBI PopSet: 1773394806) and extracted the *CYTB* sequences from our samples. These were aligned with Clustal Omega and tree was constructed with IQ-TREE (-bb 1000 -msub mitochondrial). FigTree v. 1.4.4 was used to visualize the tree.

## Supplementary material

Supplementary data are available at *Genome Biology and Evolution* online (http://www.gbe.oxfordjournals.org/).

## Supplementary Material

evac175_Supplementary_Data

## Data Availability

The raw reads and the assembled mitochondria are available in the NCBI BioProject database (http://www.ncbi.nlm.nih.gov/bioproject/) under BioProject number PRJNA827658 and GenBank accession numbers OP328298-OP328300 and OP345288-OP345340. Used commands and phylogenetic data sets are available at https://github.com/nvlain/histiotus2022.
