# Supplementary material for: Whole-genome Analysis Reveals Contrasting Relationships Among Nuclear and Mitochondrial Genomes Between Three Sympatric Bat Species
Source: Genome Biol Evol. 2022 Dec 22;15(1):evac175. doi: 10.1093/gbe/evac175 (PMC9825270; doi:10.1093/gbe/evac175)
Supplement: evac175_Supplementary_Data [file evac175_supplementary_data.zip › SuppFigures_rev2.pdf]

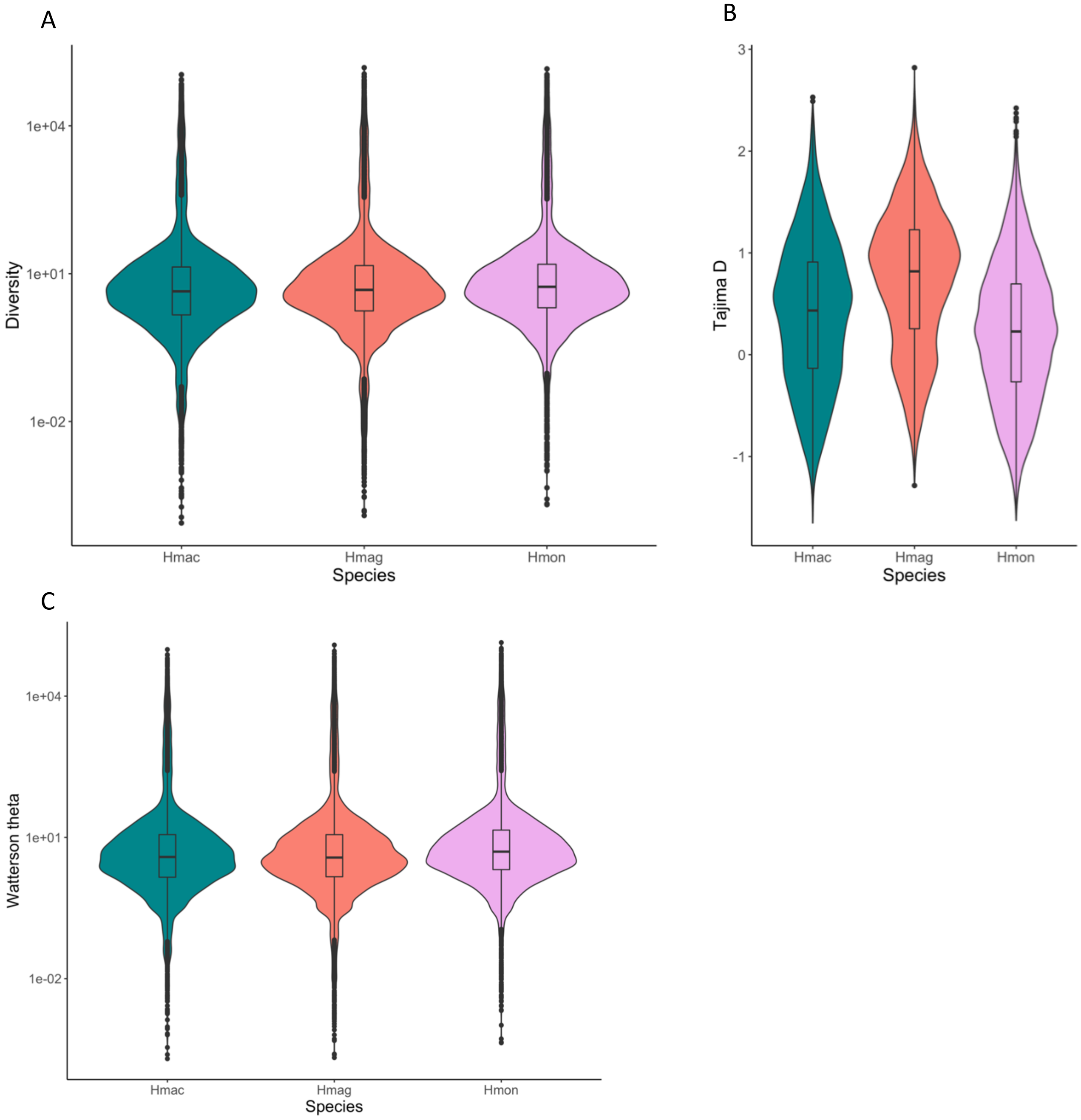

Supplementary Figure 1. Diversity indices of the *Histiotus* species a) diversity b) Tajima's D c) Watterson theta

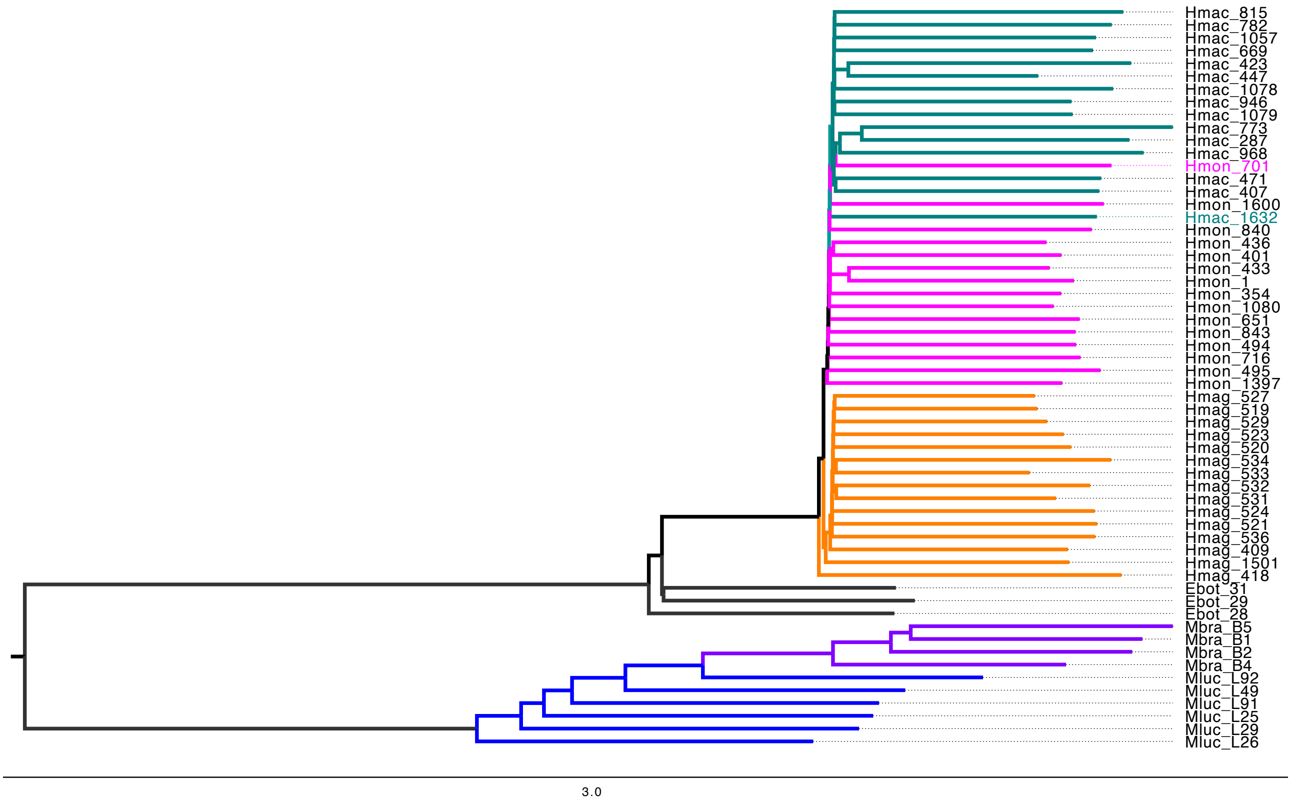

Supplementary Figure 2. Nuclear species trees derived from 9 943 522 genotype likelihood sites by using neighbor-joining method.

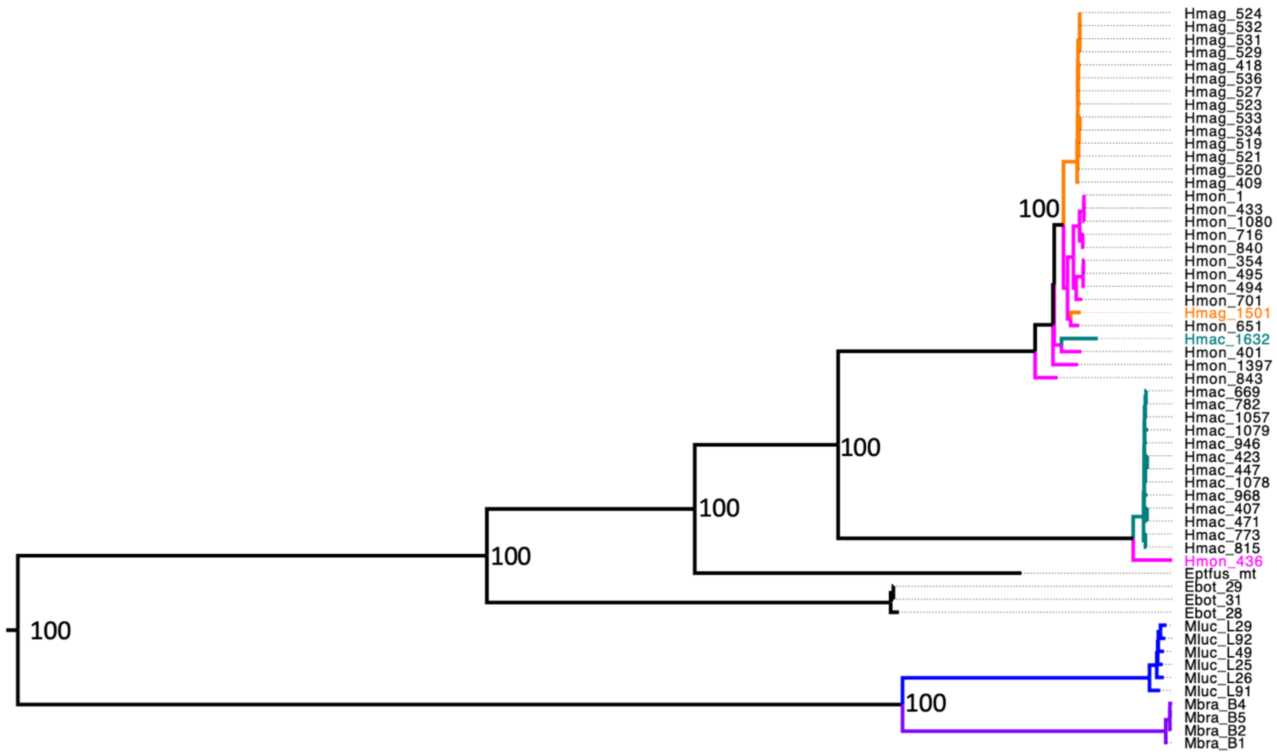

Supplementary Figure 3. Mitochondrial species consensus tree using 13 protein coding genes from IQ-TREE tree after partition and model finding. Bootstrap values given for the main nodes.

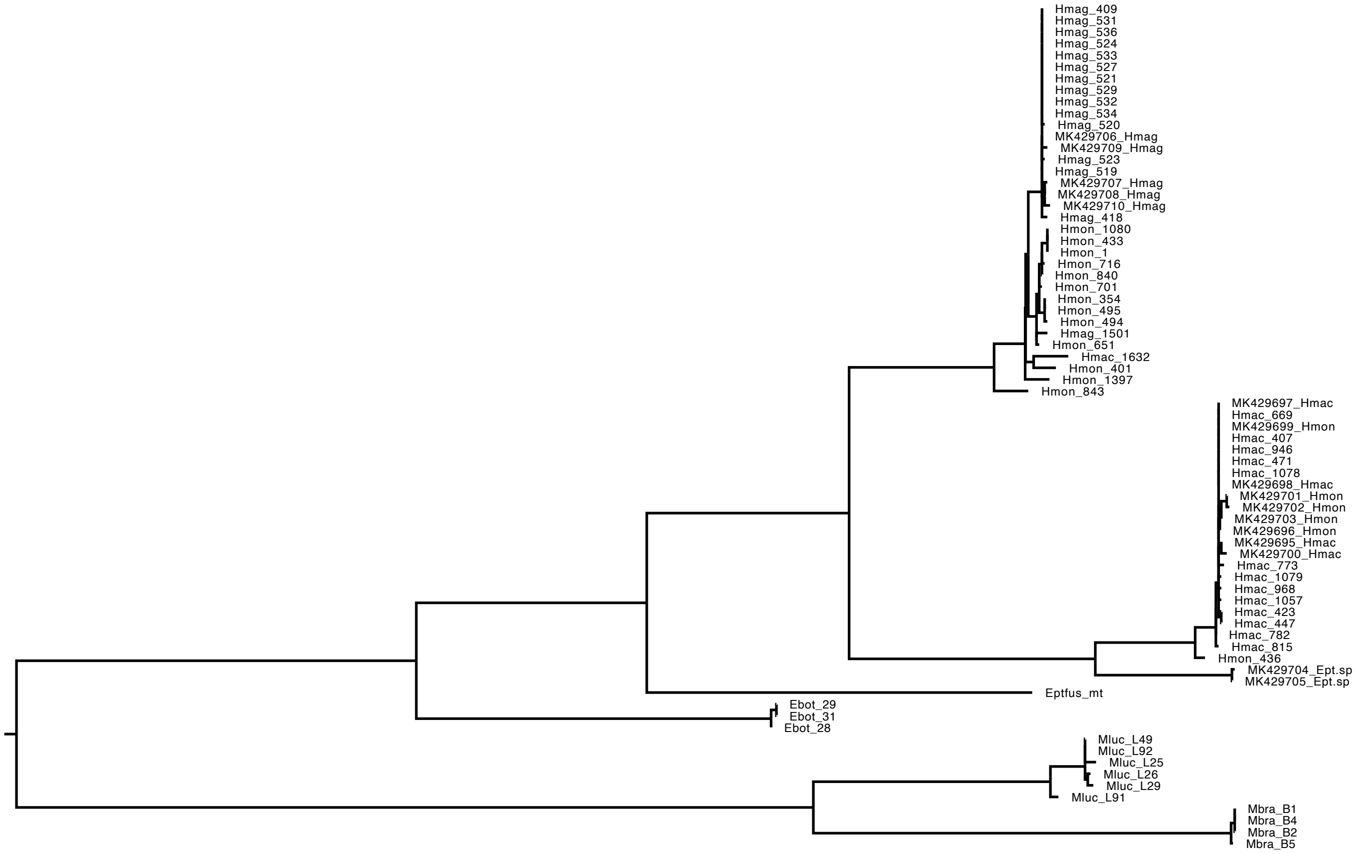

Supplementary Figure 3. Mitochondrial species tree using only *CYTB* sequences from this study and Gimenez et al. 2019 (individuals starting with "MK").
